# Endoscopic closure of a refractory gastric fistula using a novel cap-assisted poly-glycolic acid sheet application method

**DOI:** 10.1055/a-2731-6054

**Published:** 2025-11-26

**Authors:** Noriaki Sugawara, Taro Iwatsubo, Kazuki Takayama, Shun Sasaki, Akitoshi Hakoda, Kazuhiro Ota, Hiroki Nishikawa

**Affiliations:** 1 38588 Endoscopy Center, Osaka Medical and Pharmaceutical University Hospital, Osaka, Japan; 2 13010 The Second Department of Internal Medicine, Osaka Medical Pharmaceutical University, Osaka, Japan

Gastrointestinal perforations and fistulas are severe conditions requiring prompt treatment. Endoscopic closure of fistulas using a polyglycolic acid (PGA) sheet (Neoveal; Gunze Co., Kyoto, Japan) with fibrin glue is minimally invasive and effective treatment for these conditions 1 2 3 . However, for fistulas caused by peptic ulcers, it is desirable to cover not only the perforation but also the surrounding ulcerated area with a large sheet. The conventional method of PGA sheet application through the accessory channel of the endoscope for fistula closure is relatively simple; however, deploying the sheet endoscopically within the gastrointestinal tract can be time-consuming due to the difficulty in maintaining its planar shape. Patients with gastrointestinal perforations or fistulas often present in poor general conditions, necessitating rapid wound closure.

To address this challenge, we developed the “Cap-PGA method (CPM),” a novel PGA sheet application technique utilizing a cap attached to the endoscope tip ( Fig. 1 ). The CPM facilitates the release of the sheet that remains fully expanded within the gastrointestinal tract, thereby simplifying deployment and significantly reducing the procedural time. This method is also adaptable to larger sheets.

**Fig. 1:**
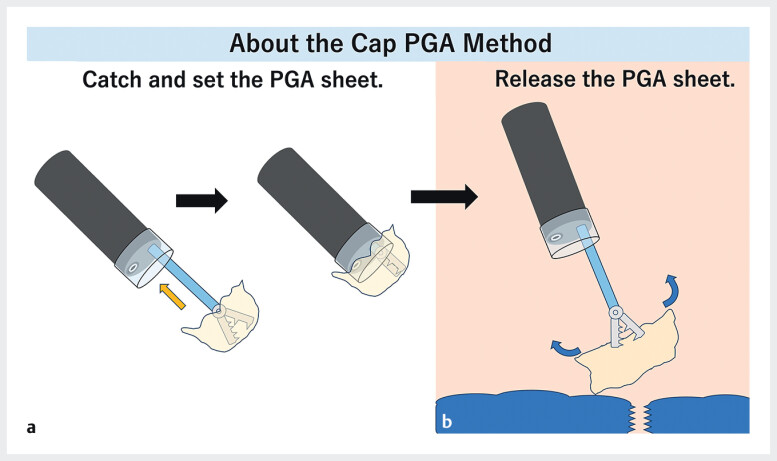
**a** The schema regarding the cap PGA method. **b** The PGA sheet is grasped with biopsy forceps inserted through the endoscope working channel and positioned to cover the tip of the scope. **c** The PGA sheet was released after endoscopic insertion into the stomach. This method enabled the sheet to be fully expanded upon deployment.

We report the case of a 71-year-old female with a refractory gastric fistula secondary to a drug-induced gastric ulcer perforation, which was successfully closed using CPM ( Video 1 ). The patient was undergoing immunosuppressive therapy. She developed a drug-induced gastric ulcer perforation, which was initially managed conservatively but resulted in a persistent fistula ( Fig. 2 ). Conventional endoscopic PGA sheet application was attempted but failed because of dislodgement. Alternatively, we attempted to use the CPM. The PGA sheet was grasped using forceps passed through the working channel of the endoscope and positioned to cover the cap attached to the scope tip. The scope was then blindly introduced into the stomach, and the sheet was released. The sheet maintained its fully expanded shape upon release, allowing for easy application to the ulcer base and fistula ( Fig. 3 , Fig. 4 ). No procedure-related complications occurred. The total procedural time from scope insertion to withdrawal was approximately 6 min. Ten days after the procedure, endoscopic contrast examination confirmed the successful closure of the fistula.

**Fig. 2:**
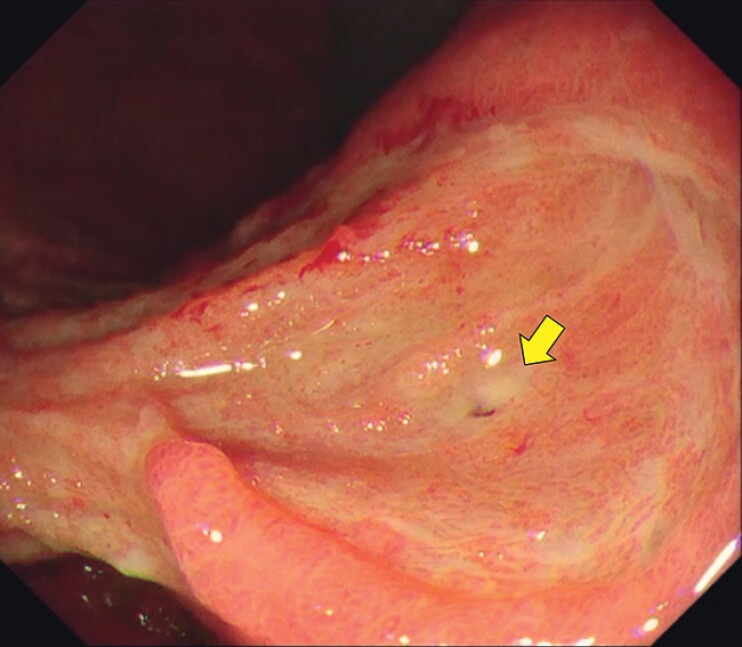
Endoscopic images showing gastric ulcers and a pinpoint fistula (yellow arrow).

**Fig. 3:**
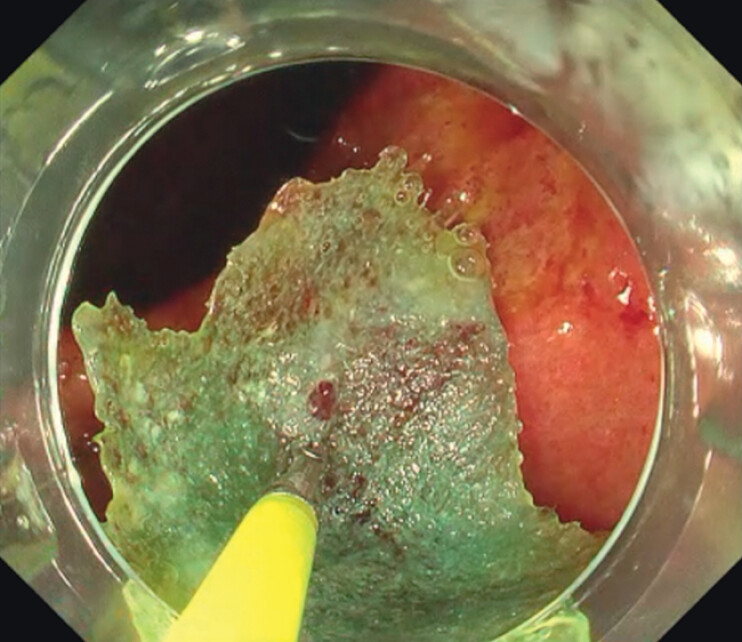
The sheet could be released in a fully expanded state within the stomach, facilitating rapid and simple application.

**Fig. 4:**
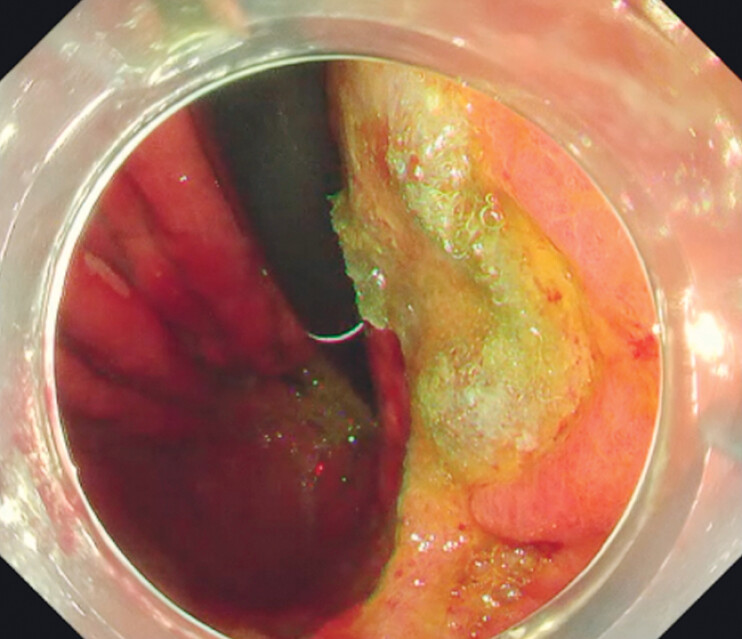
The sheet was ultimately affixed using fibrin glue, thereby completing the intervention.

The Cap-PGA method facilitates the rapid and secure endoscopic deployment of a PGA sheet for fistula closure, improving efficiency in challenging cases.Video 1

CPM allows for rapid and relatively simple coverage of fistulas with PGA sheets. This technique may be an effective treatment, especially for ulcers, fistulas, and perforations that require larger PGA sheets.

Endoscopy_UCTN_Code_TTT_1AO_2AI

## Notes

Endoscopy E-Videos https://eref.thieme.de/e-videos
*E-Videos* is an open access online section of the journal *Endoscopy* , reporting on interesting cases and new techniques in gastroenterological endoscopy. All papers include a high-quality video and are published with a Creative Commons CC-BY license. Endoscopy E-Videos qualify for HINARI discounts and waivers and eligibility is automatically checked during the submission process. We grant 100% waivers to articles whose corresponding authors are based in Group A countries and 50% waivers to those who are based in Group B countries as classified by Research4Life (see: https://www.research4life.org/access/eligibility/ ). This section has its own submission website at https://mc.manuscriptcentral.com/e-videos .
